# Minimising losses to predation during microalgae cultivation

**DOI:** 10.1007/s10811-017-1112-8

**Published:** 2017-03-10

**Authors:** Kevin J. Flynn, Philip Kenny, Aditee Mitra

**Affiliations:** Swansea University, Swansea, SA2 8PP UK

**Keywords:** Microalgae, Production, Pest control, Zooplankton, Predator-prey, Optimisation, Biofuels, Stoichiometric ecology

## Abstract

We explore approaches to minimise impacts of zooplanktonic pests upon commercial microalgal crops using system dynamics models to describe algal growth controlled by light and nutrient availability and zooplankton growth controlled by crop abundance and nutritional quality. Losses of microalgal crops are minimised when their growth is fastest and, in contrast, also when growing slowly under conditions of nutrient exhaustion. In many culture systems, however, dwindling light availability due to self-shading in dense suspensions favours slow growth under nutrient sufficiency. Such a situation improves microalgal quality as prey, enhancing zooplankton growth, and leads to rapid crop collapse. Timing of pest entry is important; crop losses are least likely in established, nutrient-exhausted microalgal communities grown for high C-content (e.g. for biofuels). A potentially useful approach is to promote a low level of P-stress that does not adversely affect microalgal growth but which produces a crop that is suboptimal for zooplankton growth.

**Electronic supplementary material:**

The online version of this article (doi:10.1007/s10811-017-1112-8) contains supplementary material, which is available to authorized users.

## Introduction

Microalgae have been proposed as a commercially important crop in support of a variety of products, ranging from biofuels to pharmaceuticals and feed-stocks for aquaculture (Greenwell et al. 2010; Milledge 2011; Borowitzka 2013). As with all crops, there is potential for production to be decreased or spoilt by the activity or presence of pests. In closed bioreactors, under laboratory conditions, there is the opportunity to exclude or control pests by using clean techniques, but in open pond systems and in large commercial bioreactor operations, there is enhanced scope for entry of pests. For microalgal crops, there are three potential pest types: contamination by other microalgae (Smith et al. 2005), infections caused by viruses which can destroy algal growth very rapidly (as noted sometimes in nature; Schroeder et al. 2003) and fungal attack such as those by chytrid fungi (Gutman et al. 2009; Strittmatter et al. 2016) and the presence of predators (Day et al. 2012). Predators may be present at very low levels in stock cultures and, depending on the growth conditions, may not normally be apparent at all (especially likely with protistan pests). In other instances, and notably in open ponds, predators of various types (protists, rotifers and crustacea such as *Daphnia* and copepods), may be introduced from the wider environment.

To date, there is no single established method successfully used to maximise microalgal production with simultaneous minimisation of crop loss through zooplanktonic predation. Cultivators of microalgae have resorted to a wide variety of strategies to control contamination. Most approaches rely on the culturing of extremophiles under highly selective growth conditions (Borowitzka 2005) with regards to pH or, in the case of marine species, salinity, with the latter having the potential double benefit of stimulating productivity while suppressing increases in invader populations (Bartley et al. 2013). Other methods can include filtration and the use of chemical pesticides (Bacellar Mendes and Vermelho 2013; Wang et al. 2013; McBride et al. 2014), although the former can only work when predators are relatively large, while imprudent use of pesticides can destroy the microalgae along with the predator (Méndez and Uribe 2012). Pulsed electric fields, intended to cause structural and functional damage to predators while leaving the microalgal cells intact, have also been suggested (Rego et al. 2015).

Other workers have proposed a more “top-down” approach to the problem by turning the hunter into the hunted with the introduction of zooplanktivorous fish into the system (Smith et al. 2010). The rationale behind such suggestions stems from the belief that monoculture states are naturally unstable; so, it is better to manage the inevitable increase in diversity by the creation of a “synthetic community” (Kazamia et al. 2012; Smith and Crews 2014). It has also been suggested that such a top-down bio-manipulation of trophic cascades may pay dividends through increased lipid production (Sturm et al. 2012). However, to preserve biochemical consistency within the crop at the point of harvest (which is usually a commercial imperative), uni-algal cultivation will most likely remain the favoured approach except perhaps for the formulation of aquaculture feeds. In view of the difficulty in applying effective predation mitigation strategies in an industrial setting, it is unsurprising that progress in this area remains slow (Chisti 2013b); this was the motivator for the current work.

The growth rate of microalgae, and the form of their biomass in biochemical terms (most basically, as indicated by their C/N/P elemental stoichiometry), is of paramount importance for commercial viability, crop production and also for the growth of grazing pests. Traditionally, a biomass C/N/P stoichiometry in accordance with the work of Redfield (1934), termed the Redfield ratio, is deemed to be optimal for microalgal growth and health (Geider and LaRoche 2002). Often, light limitation developing through self-shading affects the scope for nutrient limitation within dense microalgal populations, and in consequence, the so-called optimal N/P nutrient supply ratio does not simply, nor necessarily at all, drive balanced growth (Flynn 2010). There is a broadly linear relationship between cellular N/C and N-limiting growth rate and a strongly curvilinear relationship for cellular P/C under P-limiting growth (Elrifi and Turpin 1985; Flynn 2008); in consequence, some level of P-limitation can be incurred by microalgae without a significant impact on growth rate, nor significantly affect biochemical quality (Mayers et al. 2014).

Research on the relationship between microalgal C/N/P and growth of its natural predator, the zooplankton, indicates that prey C/N/P aligning closely with Redfield ratios best supports predator growth (Sterner and Elser 2002). Furthermore, this interaction is self-reinforcing; a predator feeding on poor quality algae (i.e. low N/C and/or low P/C) releases (regenerates) less nutrients to be re-assimilated by the remaining algae, and hence, the nutrient status of the microalgal population as prey for the grazer can deteriorate further (Mitra and Flynn 2006). For commercial exploitation, the relationship between the microalgal C/N/P and its value as a crop is most obviously divisible on whether the crop is intended for use as feedstocks that are either protein-rich (high N/C) or alternatively C-rich (high C/N, having accumulated extra C as carbohydrate and/or fatty acids). The options for optimising the rate of production for bulk biomass either for high-value chemicals, such as carotenoids and phycobilins (Borowitzka 2013), or for PUFAs and biofuel feedstocks (Fon Sing et al. 2013) are thus largely mutually exclusive. As a high microalgal C/N is associated with low growth rates and hence low biomass productivity, maximising production of C-rich products requires careful selection of microalgal physiology (Flynn et al. 2010, 2012) and also careful management of bioreactors with respect to their design, lighting and nutrient loading and harvesting or bioreactor dilution rates (Kenny and Flynn 2015).

For commercial growth of microalgae, the organisms are grown to high densities (with dry weight biomass concentration typically between 0.1 and 0.5 kg m^−3^; Chisti 2007; Ozkan et al. 2012) and supplied with high nutrient loads. Under these conditions, light limitation of growth is likely (Richmond 2013) so that growth reactors contain dense suspensions of potentially high-quality prey for zooplankton. Further, in contrast to the situation in nature, prey availability is not limiting for zooplankton growth. If the reactor is operated in a continuous dilution (chemostat-like) mode, then conditions may further conspire to favour the growth of the zooplankton over the microalgae, as biomass-specific grazing rates typically exceed biomass-specific growth rates of the crop (Hansen et al. 1997). However, if the crop is grown for a high C-content (high C/N), then there is scope to minimise losses through the formation of a crop that is intrinsically of low nutrient quality for predators (Sterner and Elser 2002; Mitra and Flynn 2005).

This work uses computational stoichiometric ecology to identify approaches (forgoing genetic modification, GM, of the crop) for the control of predation in commercial microalgal cultivation systems through manipulation of factors such as nutrient regimes and culture harvesting.

## Methods

### System configuration

**Fig. 1:**
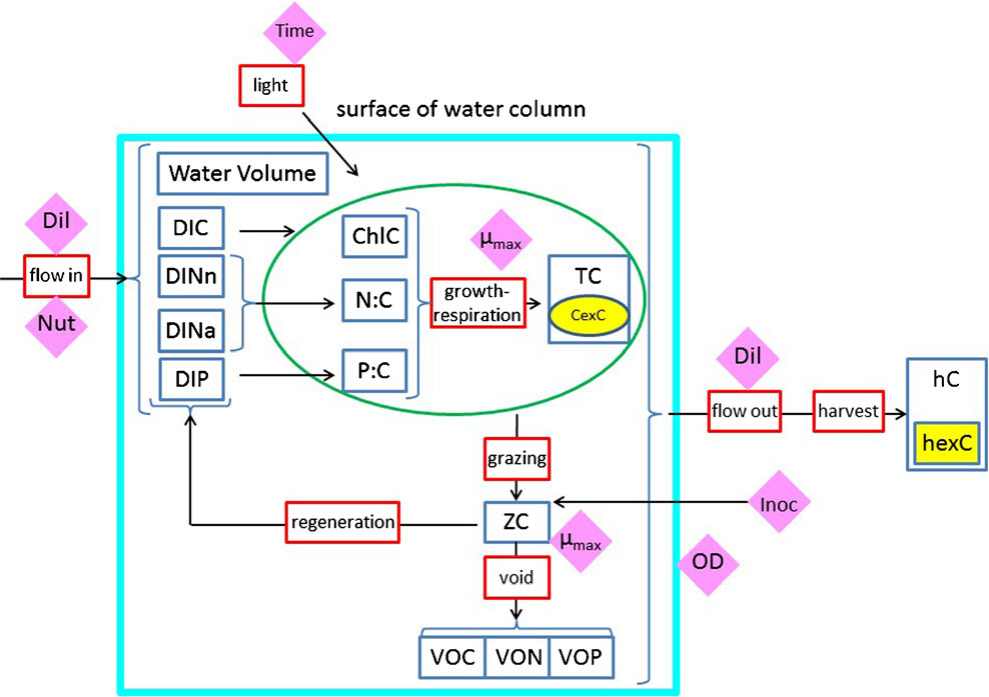
Schematic of the model. Items within *dark blue boxes* are state variables defined in the model. Nutrients for algal consumption include dissolved inorganic C (DIC), ammonium (DINa) and nitrate (DINn) and phosphate (DIP). The increase in microalgal C-biomass (TC) is a function of the nutrient status (N/C and P/C) of the cells, the status of the photosystems (ChlC, which is itself a function of nutrient status and photoacclimation) and light availability. Light reaching the microalgae depends on surface irradiance, light absorbance by water and the pigmented microalgae and operation depth (OD; pond depth or diameter of a bioreactor tube). A proportion of algal biomass (TC) is C-rich storage products (carbohydrate + lipid; CexC). Zooplankton predator C-biomass (ZC) increases through grazing, with part of the ingestion biomass being voided as organic C/N/P, and part regenerated as nutrients for re-assimilation by the microalgae. The efficiency of grazing (conversion of TC to ZC) depends on algal N/C and P/C, according to stoichiometric rules. Harvesting contributes to harvested biomass (hC), including harvested material for biofuels (hexC). Indicated by *diamonds* are the key parameters explored in the simulations: *Dil* dilution rate and harvesting frequency, *Inoc* inoculation of the system with zooplankton, *Nut* nutrient concentration, *OD* operational depth, *Time* day and hour, *μ*_*max*_ maximum growth rate of the algae and of the zooplankton predator. Light available for microalgal photosynthesis is a function of that surface irradiance over the day-night cycle, absorbance by the algal suspension with reference to OD, TC and ChlC (the latter a function of NC and of light availability via photoacclimation). The value of hexC depends on TC and CexC, which in turn relates to Dil and Nut, such that growth rate is optimised while N/C is low (noting that N/C is linearly related to N-limited growth potential)

The investigation described here exploited the use of dynamic variable stoichiometric models (i.e. for algae, variable C/N/P/Chl). This is in recognition of the importance of simulating variable stoichiometry not only for describing biomass and carbohydrate/lipid production by microalgae (Kenny and Flynn 2015) but also for predator-prey interactions (Sterner and Elser 2002; Mitra and Flynn 2005). A schematic of the model is given in Fig. 1; a full description, with equations and examples of prior usage to establish the models providence, is given in the Supplementary material (Appendix_A_Model_Info.xls).

The microalgal sub-model was similar to that used in our studies on optimisation of microalgal production (Flynn et al. 2012; Kenny and Flynn 2015, 2016). Thus, microalgal growth was described using a mechanistic, acclimative, variable stoichiometric model of microalgal physiology. Changes in microalgal C/N/P/Chl occur with acclimation in response to changes in nutrient and light availability. With nutrient exhaustion, especially of nitrogen (N), excess carbon (C) is accumulated (i.e. cellular C/N increases; N/C declines). Whether that excess C accumulates in reality as carbohydrate and/or fatty acids depends on the taxonomic characteristics of the organism. This particular microalgal model has been used to describe the growth dynamics of many different species under different situations (Appendix_A_Model_Info.xls online). A demonstration of the model operating against a published data set for a real reactor system (Quinn et al. 2012) is shown in Kenny and Flynn (2016).

To this original microalgal-bioreactor model, we added a model describing the growth dynamics of zooplankton (Mitra 2006). That zooplankton model has been previously configured to simulate the growth of both microzooplankton (protists) and mesozooplankton (crustacea) and has been used under various scenarios (Mitra and Flynn 2006; Mitra et al. 2007, 2014 and references therein; see Appendix_A_Model_Info.xls).

The physiological descriptions of the microalgae and zooplankton were as used in Flynn et al. (2012). The maximum growth rate of the microalgae enabled a division per day (i.e. μ = 0.693 day^−1^) under the 12:12-h light/dark cycle employed. This growth rate is consistent with the enzyme characteristics and cellular activity of RuBisCO (the enzyme that fixes CO_2_), which may enable a maximum growth rate in continuous illumination approaching two divisions per day (Flynn and Raven 2017). The zooplankton were simulated with either a growth rate equivalent to a doubling per day, or two doublings per day. The maximum zooplankton assimilation efficiency was set at 0.75, but this decreased with deterioration in prey quality with reference to the prey and predator N/C and P/C ratios, using which ever was the lower of preyNC/predNC or preyPC/predPC (Mitra 2006). The result of this linkage is an increasingly poor trophic transfer from the prey to the predator as the prey quality declines (i.e. as microalgal N/C and/or P/C falls); this description accords with empirical evidence (e.g. Sterner et al. 1993; Flynn et al. 1996; Young et al. 1997).

The whole model, describing microalgae and zooplankton growth within a description of the physico-chemical environment of a bioreactor or pond, was run with an integration step size of 11.25 min. The growth environment included descriptions of the supply of inorganic dissolved N and P nutrients (and their recycling with any zooplankton activity), of light (changing in its availability to the microalgae as a function of the self-shading activity of the plankton biomass, as well as over a 12:12-h light/dark cycle), of bioreactor depth (assuming the typical homogenous mixing of the contents) and of harvesting rates (see below). The default primary limiting nutrient concentration was 440 μM inorganic N (which is half the concentration of the classic f/2 marine algal growth medium; Guillard 1975). We have previously shown that this nutrient provision allows potential nutrient exhaustion under non-light limiting conditions over a range of the shallower optical paths (“depths”) typically used in photobioreactors (Kenny and Flynn 2015). In addition to this, P was supplied at either the Redfield ratio with N (mole ratio for N/P of 16) or at twice that ratio (N/P of 32) to enable a potentially moderate level of co-P stress in the microalgae. Growth at a nutrient N/P of 32 appears not to be deleterious to phytoplankton physiology or fatty acid production (Mayers et al. 2014), consistent with the distinct curvi-linear (quota) relationship between cellular P/C and growth rate (Flynn 2008).

### Harvesting techniques

We explored different crop harvesting approaches. Harvesting can be continuous (akin to a chemostat operation, i.e. described by a dilution rate) or discontinuous (as in a batch growth system) with some fraction of the culture harvested every few days. However, in reality, in preparation for developing systems for either approach, a culture must first be grown up in what amounts to a batch growth system. Accordingly, here, growth was simulated from a low inoculum into a low-dilution continuous dilution system; this gave a period of nutrient-replete exponential growth (batch-culture like) prior to steady-state (chemostat-like) growth but without risk of an abrupt nutrient stress than may damage the crop.

### Introducing the zooplankton

For the simulations presented here, the introduction of the zooplankton predator was considered to occur either concurrent with the microalgae at the start of the growth cycle (i.e. as a contamination in the original inoculum), or after establishment of steady-state conditions (here contamination occurred at day 20 of the simulation, into a well-developed algal system). In both instances, it was assumed that the initial zooplankton biomass was only 0.05% of that of the microalgae. If one considers a predator with a size (volume) of at least 10 times that of its prey (as would typically be the case—Hansen et al. 1997), then numerically, this level of zooplankton contamination would equate to only 0.005% (1 in 20,000). Such contamination levels would likely go unnoticed in routine microscope sample examination of cell counts of the microalgae (Day et al. 2012).

### Presentation of results

Results are given in terms of the rates of areal biomass production (AP; gC m^−2^ day^−1^) and areal production of C-rich components (fatty acids and/or carbohydrate; AXP; gC m^−2^ day^−1^), as well as volumetric production (VP; gC m^−3^ day^−1^). Areal productivity takes into account VP and bioreactor depth (m). Table 1 gives a summary of the simulated test conditions and their associated figures. Here, we highlight specific examples of simulations; additional results (as indicated in Table 1) are provided in the Supplementary Material Appendix B and referenced in the format Fig. S*x*. It should be noted that maximum (standing stock) biomass levels reflect a balance of growth rate set against dilution rate. Emphasis here is placed on production rate (the commercial imperative) and not upon terminal biomass concentration.

**Table 1:** Summary of simulation conditions and plot locations

Nutrient mole N/P	Dilution rate (day^−1^)	Areal biomass: algal and zooplankton	Volumetric biomass: algal and zooplankton	Algal N/C and N/P	Algal AP and AXP	Zooplankton growth and ingestion rates	Zoo-plankton AE and GGE
16	0.1	Fig. S1					
32	0.1	Fig. S2					
16	0.3	Fig. 2	Fig. S5	Fig. 4	Fig. S7	Fig. S9	Fig. S11
32	0.3	Fig. 3	Fig. S6	Fig. 5	Fig. S8	Fig. S10	Fig. S12
16	0.5	Fig. S3					
32	0.5	Fig. S4					

Plots given in the Supplementary material (Appendix B online) are indicated by Fig. S*x*. In all instances, the simulations were performed over a range of reactor depths from 0.025 to 0.5 m

*AP* areal biomass production, *AXP* areal production of C-rich components (fatty acids and/or carbohydrate), *AE* assimilation efficiency (proportion of material ingested by zooplankton that is not voided), *GGE* zooplankton gross growth efficiency (ratio of growth rate to ingestion rate)

## Results

### Control systems, with no pest introduction

**Fig. 2:**
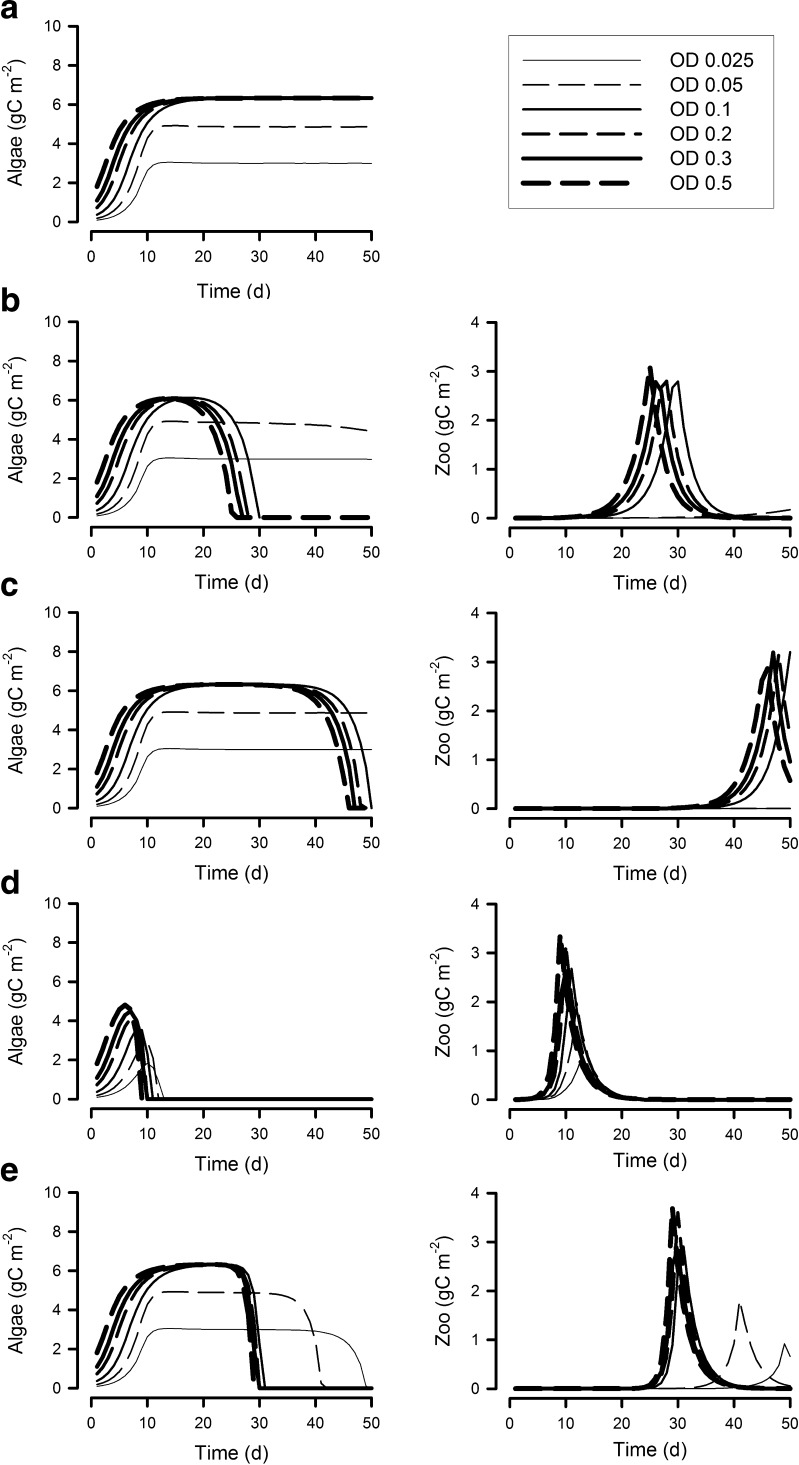
Areal biomass of algae and of the zooplankton contaminant when grown at 6 different reactor operation depths (OD; 0.025–0.5 m), with the supply nutrient mole ratio N/P at 16 and a dilution rate of 0.3 day^−1^. **a** No contamination. **b** Contamination at 0 day. **c** Contamination at 20 days. **d** Contamination at 0 day with fast growing zooplankton. **e** Contamination at 20 days with fast growing zooplankton

**Fig. 3:**
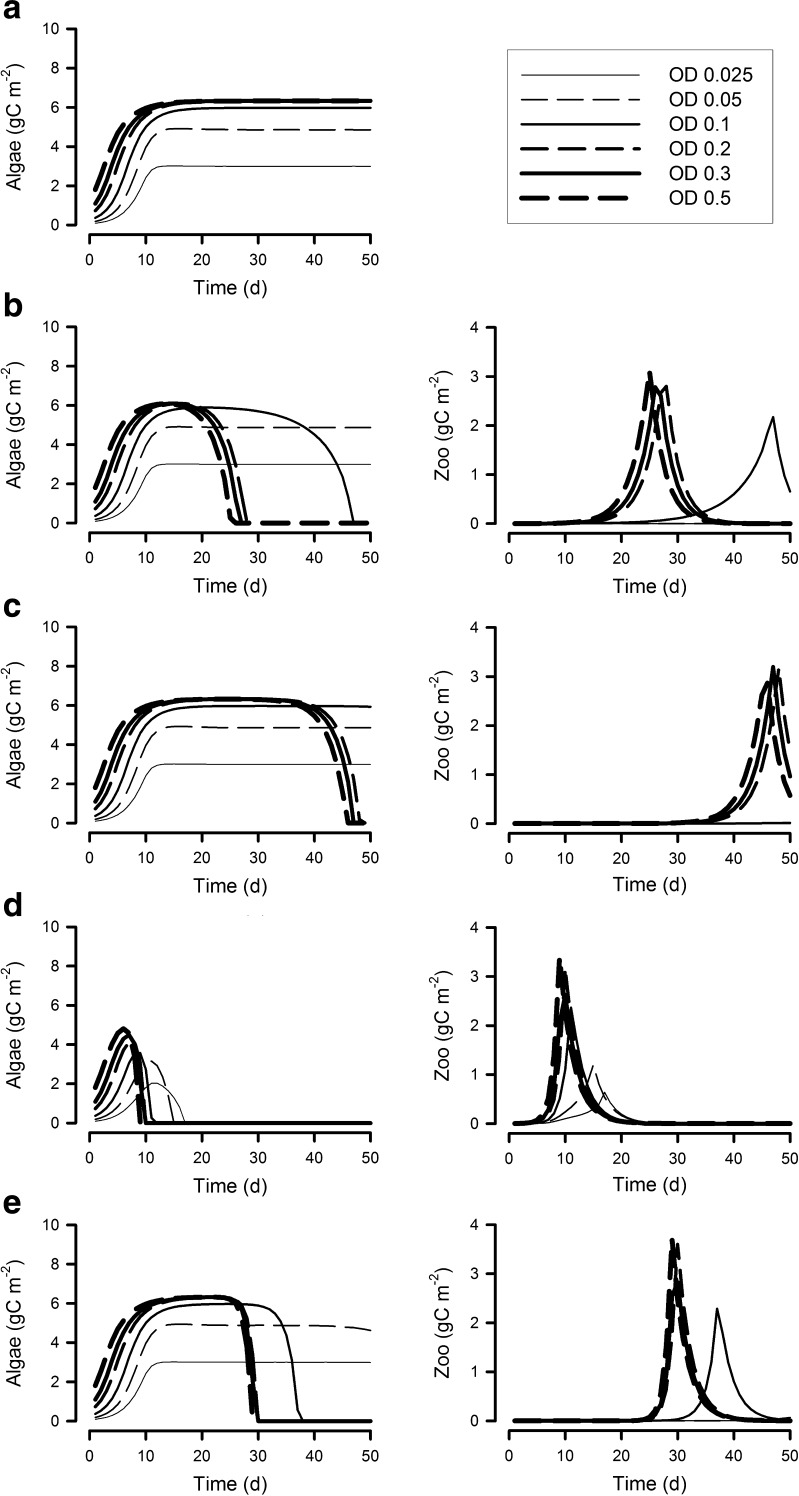
As Fig. 2 but with a supply nutrient mole ratio N/P of 32. **a** No contamination. **b** Contamination at 0 day. **c** Contamination at 20 days. **d** Contamination at 0 day with fast growing zooplankton. **e** Contamination at 20 days with fast growing zooplankton

Figures 2a and 3a show microalgal growth in systems of different depths (0.025 to 0.5 m), with different N/P nutrient supply ratios and different dilution rates (panels (a) in Figs. S1–S4). There are no substantial differences in the microalgal growth rates between systems supplied with nutrients at the higher N/P (N/P = 32) versus the lower, “optimal”, N/P of 16. Peak areal biomass (i.e. as gC m^−2^) decreases with increasing dilution rate (Figs. 2a and 3a versus Figs. S1–S4). The volumetric biomass density of microalgae exceeded 100 g C m^−3^ in shallow systems operating at a dilution rate of 0.3 day^−1^ (Figs. S5a and S6a).

**Fig. 4:**
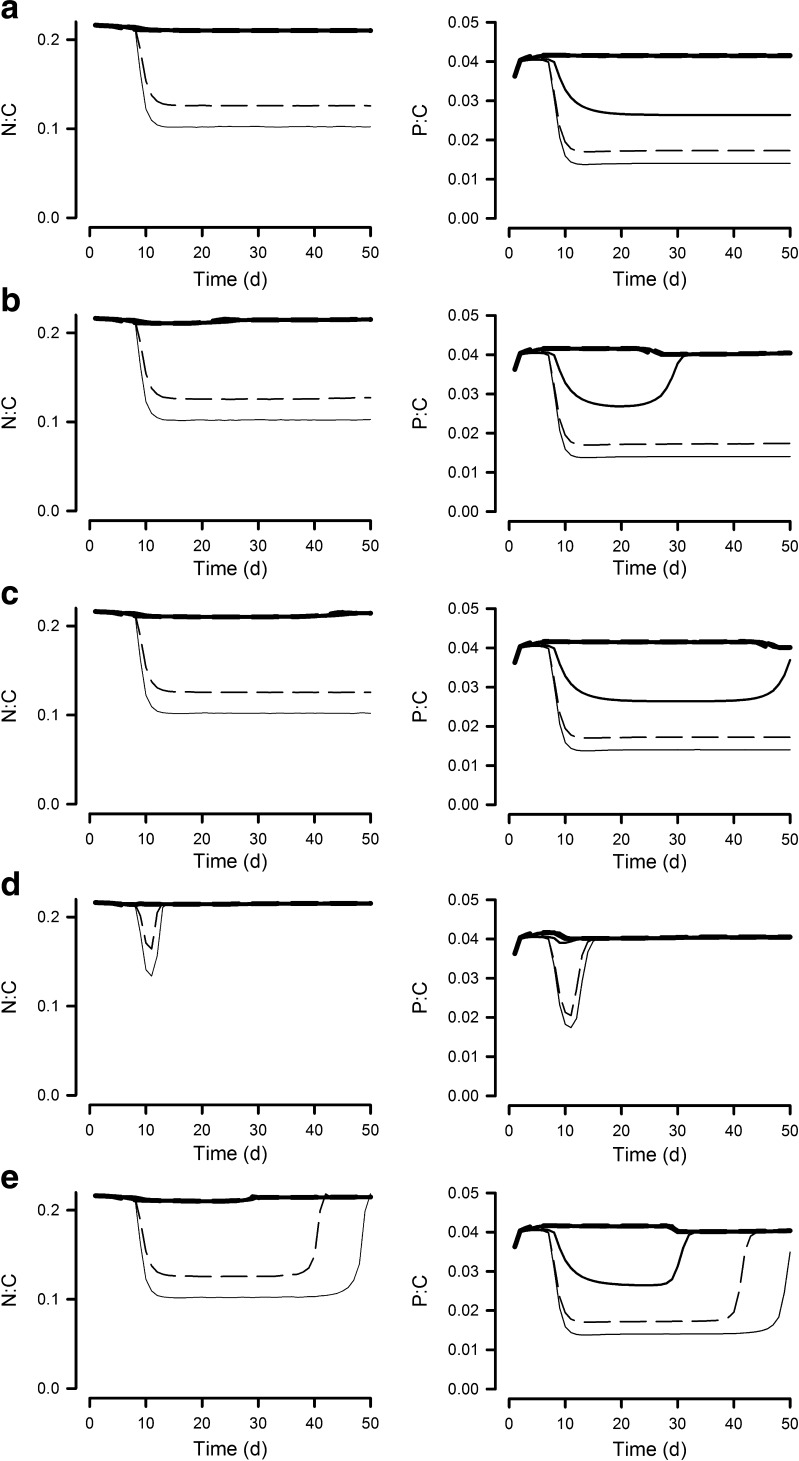
Algal mass ratios of N/C and P/C when grown at 6 different reactor operational depths (OD; 0.025–0.5 m), with the supply nutrient mole ratio N/P at 16 and a dilution rate of 0.3 day^−1^. Cf. Fig. 2 for biomass and legend to line types. **a** No contamination. **b** Contamination at 0 day. **c** Contamination at 20 days. **d** Contamination at 0 day with fast growing zooplankton. **e** Contamination at 20 days with fast growing zooplankton

**Fig. 5:**
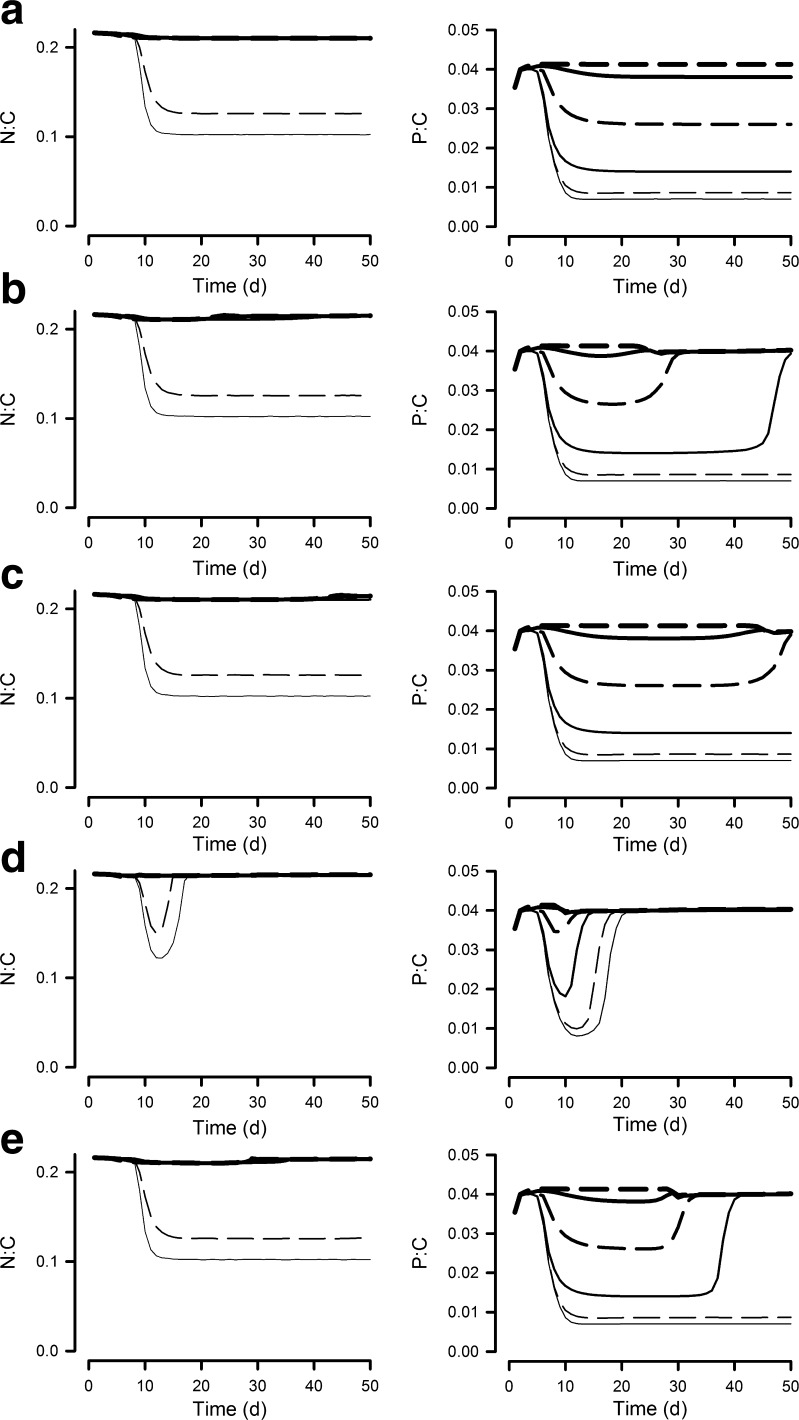
As Fig. 4, but with a supply nutrient mole N/P of 32. Cf. Fig. 3 for biomass and legend to line types. **a** No contamination. **b** Contamination at 0 day. **c** Contamination at 20 days. **d** Contamination at 0 day with fast growing zooplankton. **e** Contamination at 20 days with fast growing zooplankton

Microalgal N/C was high (i.e. indicative of N-replete and/or light-limiting conditions) in all reactor systems except those with depths <0.1 m (Figs. 4 and 5). Accordingly, significant areal production of high-C metabolites, such as could be used for lipid nutrition or biofuels, only occurs in these shallow systems (AXP; Fig. S7a) where significant nutrient limitation can develop. Nutrient supply N/P does not greatly affect cellular N/C (Figs. 4 and 5) nor AXP (Figs. S7 and S8). Microalgal P/C in the N/P = 16 systems remains high at depths >0.2 m (Fig. 4), but in N/P = 32 systems (Fig. 5), only the deepest system (depth 0.5 m) enables an elevated microalgal P/C with shallower systems enabling the development of P-stress.

### Systems contaminated with zooplanktonic pests

In the simulations, we considered two scenarios, one with contamination by zooplankton at the point of inoculation, and the other into an established crop. When contaminated with zooplankton with a maximum growth rate similar to that of the microalgae at the start of the system operation (Figs. 2b and 3b; Figs. S1–S4), there was no effective grazing loss in the shallow systems (0.025 and 0.05 m deep) when supplied with a low nutrient N/P. This is because these systems contained microalgae with a C/N/P stoichiometry that makes them a poor food source for zooplankton growth. In the high N/P systems (Figs. 3, S2 and S4), which produce microalgae with low P/C (Fig. 5), microalgal losses in the 0.1-m-deep system were also slower, with low grazing pressure (Figs. S9 and S10) and poor zooplankton assimilation and growth efficiencies (Figs. S11 and S12).

Contamination of established systems is less likely to lead to crop losses than an initial contamination event (panels c versus panels(b in Figs. 2 and 3; Figs. S1–S4), provided that the system is shallow enough for nutrient limitation of microalgal growth to develop, and/or where the resultant zooplankton growth rates are slower than the system dilution rate (panels c in Figs. S3 and S4). The likelihood of some level of grazing resistance is enhanced in shallow depths by the P-depletion that developed in high N/P systems, which resulted in a depressed algal P/C (Fig. 3 , S1, S2, S4 and S6); because of the resultant poor food quality, zooplankton in such systems have poor assimilation and growth efficiencies (Figs. S11 and S12).

Contamination with fast growing zooplankton leads to a more rapid demise of the microalgal crop; this is catastrophic when it occurs at the start of the culture process, when neither light nor nutrients are limiting and hence good food quality is assured (Figs. 2d and 3d; Figs. S1–S4). However, when contamination occurs into established shallow systems with a moderate system dilution rate (Figs. 2e and 3e; Figs. S1–S4), then microalgal losses are low. This is especially obvious with a high N/P medium (Fig. 3), where the poor P/C of the microalgae (i.e. poor quality food) restricts zooplankton growth to rates similar to, or below, the dilution rates of the systems (Figs. S9 and S10).

In our simulations, a high microalgal N/C ratio is maintained for depths >0.05 m and a high microalgal P/C for depths >0.2 m (Figs. 4 and 5), regardless of the timing of pest entry. These systems are more likely to support effective feeding by zooplankton, with higher assimilation and growth efficiencies (Figs. S11 and S12), and thence higher pest growth rates (Figs. S9 and S10).

## Discussion

### Control systems, with no pest introduction

Our simulated microalgal biomass values are comparable to peak concentrations measured in commercial open ponds (Ozkan et al. 2012) assuming a conversion of C-biomass to dry weight, C/dw, between 0.3 and 0.5 (Heymans 2001; Geider and LaRoche 2002). Areal productivity levels, peaking at 2 gC m^−2^ day^−1^ and equating to 7.3 tC ha^−1^ year^−1^, fall in the upper half of rates of the rate of 10–30 t dry weight ha^−1^ year^−1^ reported for real systems (Garcia-Gonzalez et al. 2003; Jiménez et al. 2003; Crowe et al. 2012), again assuming a value for C/dw between 0.3 and 0.5. The simulations of microalgal growth thus give results consistent with expectations (see also Kenny and Flynn 2016).

### Systems contaminated with zooplanktonic pests

The outcome of zooplankton-microalgal predator-prey interactions is dictated by the balance of growth and loss rates of both parties. Growth of the microalgal prey requires adequate nutrients and light, but as the formation of algal biomass increases at higher nutrient supply concentrations so does the likelihood of self-shading which leads to light limitation of photosynthesis. Loss of the microalgal crop is intrinsically related to the growth of the zooplanktonic predator which in turn depends on the nutritional quality and quantity of the available prey. In nature, both quantity and quality of prey affect zooplankton grazing. However, in artificial systems where microalgae are grown for biomass production, prey quality is the overriding issue. It is important to note that prey quality in this context need not equate to commercial crop quality; a microalgal crop grown for high lipid content will have a high C/N and thus constitute poor food for a predator (Sterner and Elser 2002; Mitra and Flynn 2005). In theory, genetic modification (GM) approaches could be used to configure microalgae that are unpalatable to zooplanktonic pest. Indeed, an analysis on the optimal configuration of GM-microalgae for biofuels and lipid production describes an organism that is coincidentally very poor feed for zooplankton (Flynn et al. 2012). While outwardly a win-win situation, the (inevitable) escape of such a GM organism from large-scale open ponds into the wild would carry a very real risk of generating a harmful algal species *par excellence*, being able to grow rapidly using little nutrient and be ungrazable (Flynn et al. 2012). For this reason, here we consider exploiting stoichiometric ecology to control pest growth.

The greatest risk from zooplankton pests is at the initial phases of crop growth, when the microalgal biomass density is relatively low (though far above levels likely to limit zooplankton grazing rates—Hansen et al. 1997) and hence neither light nor nutrients are limiting for algal growth. Microalgae in such situations are typically of good feed value for predators. Contamination of established systems is less likely to lead to crop losses if the cultivation system is shallow enough and the nutrient loading low enough to enable nutrient limitation of microalgal growth to develop. Minimising risks is improved further if a high N/P nutrient regime is operated allowing some level of P-depletion to develop, depressing algal P/C (Figs. 3, S1, S2, S4 and S6). High dilution systems populated by microalgae with growth rates that are higher relative than that of their predators are also less susceptible to zooplankton attack (Moheimani and Borowitzka 2006). However, contamination with fast growing zooplankton lessens the likelihood of crop survival whenever that contamination should occur.

To optimise production of biofuels or lipids, shallow culture systems populated with microalgae grown into nutrient limitation at a low fraction of their maximum possible growth rate (μ_max_) are required (Kenny and Flynn 2015). The higher the microalgal μ_max_, the better, as the system can also be operated at a higher absolute dilution rate and this then also washes out any zooplankton. Hence, to minimise crop losses through predation, a balance needs to be struck with bioreactor dilution rates. A slow diluted, light limiting system will favour predators. Conversely, a high dilution rate system configured for high production rates of a C-rich crop appears of lesser susceptibility to predators because the crop is of poor value as food and the elevated dilution rate then exceeds the zooplankton growth rate, washing out the pest. To illustrate this, compare the high peaks of slow-growing zooplankton contamination in Fig. S1b and S1c (where dilution = 0.1 day^−1^) to the fast dilution (0.5 day^−1^) situation in Fig. S3b and S3c; in the latter, zooplankton biomass only starts to rise very late in the simulation period.

### Manipulating the systems to commercial advantage

Setting the best culture conditions to attain optimal chemical composition and elemental C/N/P for commercial growth of microalgae is challenging. For instance, any naturally lit system is susceptible to diel and weather-induced changes in irradiance levels that pose a serious risk to production. This is not solely due to a decrease in growth rate, but because during periods of lowered irradiance, there is an attendant improvement in food quality resulting from changes in microalgal C/N/P. Hence, decreased illumination over a significant period of a day (due to cloud cover, for example) may be expected to enhance the likelihood of grazer control due to a combination of a decreased growth rate of the phototroph, coupled with a concomitant improvement in prey nutritional quality as microalgal N/C and P/C increases. Thus, in studies by Sterner et al. (1998) and Urabe et al. (2002), the balance of light and nutrient limitation affected whether the stoichiometric quality of the microalgae remained low under high light, suppressing zooplankton growth, or was improved under low light conditions, where the zooplankton quickly dominated the microalga, *Scenedesmus*.

If prey are of good nutritional quality (i.e. microalgal N/C and P/C broadly similar to that of their predator), then grazing and assimilation efficiency are expected to be optimal (Sterner and Elser 2002). Through a positive feedback loop, the nutrients regenerated by the zooplankton enhance the nutrient status of their prey and the predator population grows rapidly; the microalgal crop is thence removed rapidly as the well-fed zooplankton proliferate. However, if microalgal N/C and/or P/C become decreased due to nutrient exhaustion, then trophic transfer to the grazer is less effective; nutrient regeneration by the zooplankton is decreased or even stalled and the crop remains of poor nutritional status. Such effects on grazer activity were explored by Hessen et al. (2002) under a variety of nutrient/light conditions. When zooplankton were introduced to a stable algae culture, an increase in P-availability combined with low light levels increased the algal P/C and the zooplankton soon dominated. Conversely, zooplankton growth was slowest under high light/low P conditions, and hence, algal loss was low due to poor grazing (Hessen et al. 2002). The effects of such positive feedback processes have been demonstrated using models (Mitra and Flynn 2006). The approach needed in commercial microalgal production is to exploit these ecological features to advantage. That is relatively easy if the crop is grown for high-C products, as such microalgae are naturally poor prey. But what if the crop is grown for high protein content?

Recent evidence indicates that biomass and biochemical production by microalgal crops may be maintained when the microalgae are grown to N/P ratios higher than the Redfield N/P mole ratio of 16 (Mayers et al. 2014). This is of immediate benefit because it decreases the demand for phosphorus fertiliser—an expensive and dwindling resource (Elser and Bennett 2011; Chisti 2013a). However, there is an additional benefit because growth of zooplankton pests appears to be affected more by a lack of P (Hessen et al. 2002) than are the microalgae, providing a more grazer-resistant crop (Fig. 2 vs 3). In the simulations run here, the stoichiometric interactions between predator and prey were, in accordance with simple stoichiometric ecology (Sterner and Elser 2002), those directly and simply (linearly) related to differences in elemental C/N/P. In reality, deviations in C/N/P in phototrophs are often associated with other biochemical events such as the accumulation of secondary metabolites which are distasteful to grazers, if not potent toxins. Being stressed by P, rather than N, is most closely allied to accumulation of secondary metabolites that are noxious to zooplankton (Granéli and Flynn 2006). N-stress may also produce microalgae that are distasteful to zooplankton (Flynn et al. 1996) even though they may not be classed as noxious in a human nutrition context. Given that zooplankton grazing is most damaging when the prey (the microalgal crop) has a well-balanced C/N/P, it is fortuitous that many products of value from microalgae are derived from high-C metabolites (Greenwell et al. 2010) which are products of cells with elevated C/N and that minor changes in algal C/P may not be damaging to crop production (Mayers et al. 2014). Interestingly, P-limitation also limits chytrid parasitic infections of microalgae (Bruning 1991). Coupled with the projected increase in the cost of P-fertilisers, there appear sound reasons for minimising the addition of P in all microalgal culture systems.

## Electronic supplementary material

ESM 1 (XLSX 158 kb)

ESM 2 (PDF 475 kb)
