# Supplementary material for: Minimising losses to predation during microalgae cultivation
Source: J Appl Phycol. 2017 Mar 10;29(4):1829–40. doi: 10.1007/s10811-017-1112-8 (PMC5514209; doi:10.1007/s10811-017-1112-8)
Supplement: Supplementary file 2 — (PDF 475 kb) [file 10811_2017_1112_MOESM2_ESM.pdf]

Supplementary material

Minimising losses to predation during microalgae cultivation

Kevin J Flynn, Philip Kenny, Aditee Mitra

Additional figures. See also Table 1, and ESM file Appendix_A_Model_Info.xls

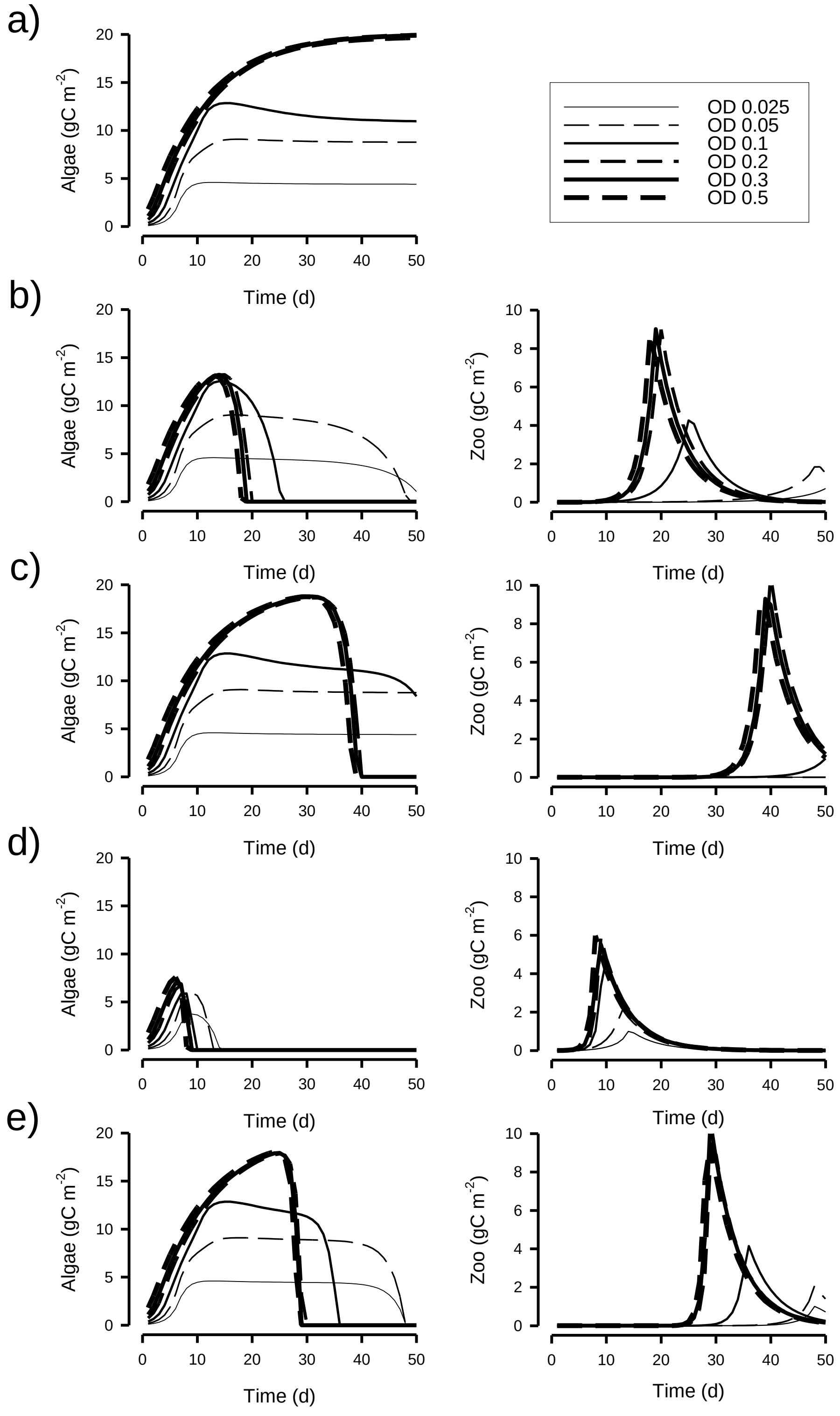

Fig. S1. Areal biomass of algae and a zooplankton contaminant when grown at 6 different operation depths (OD; 0.025 – 0.5 m), with the supply nutrient mole ratio N:P at 16 and a dilution rate of $0.1d^{-1}$. a) No contamination. b) Contamination at 0d. c) Contamination at 20d. d) Contamination at 0d with fast growing zooplankton. e) Contamination at 20d with fast growing zooplankton.

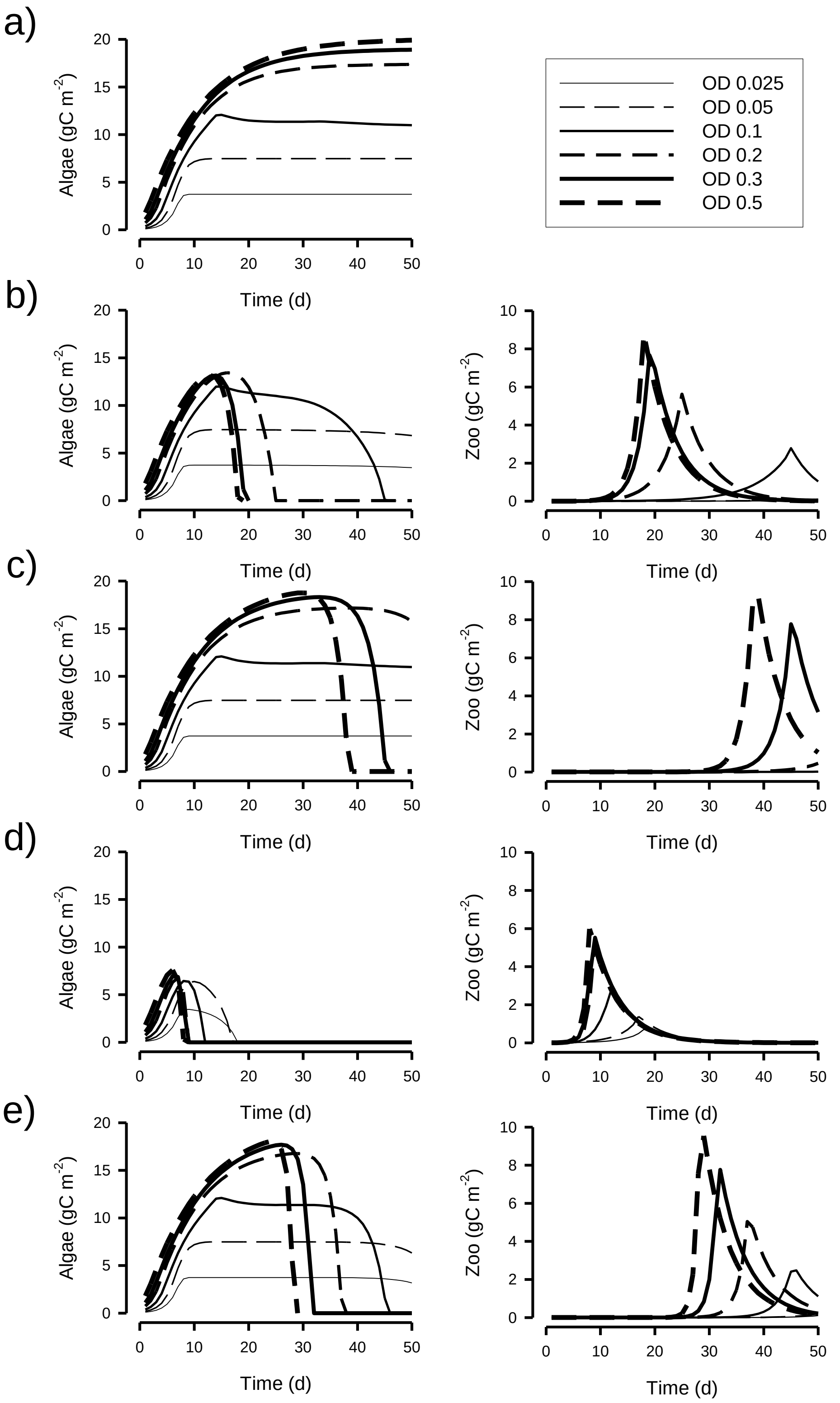

Fig. S2. Areal biomass, as Fig.S1, at 6 different operation depths (OD; 0.025 – 0.5 m), with a dilution rate 0.1 d^{-1}, but with the supply nutrient mole ratio N:P at 32. a) No contamination. b) Contamination at 0d. c) Contamination at 20d. d) Contamination at 0d with fast growing zooplankton. e) Contamination at 20d with fast growing zooplankton.

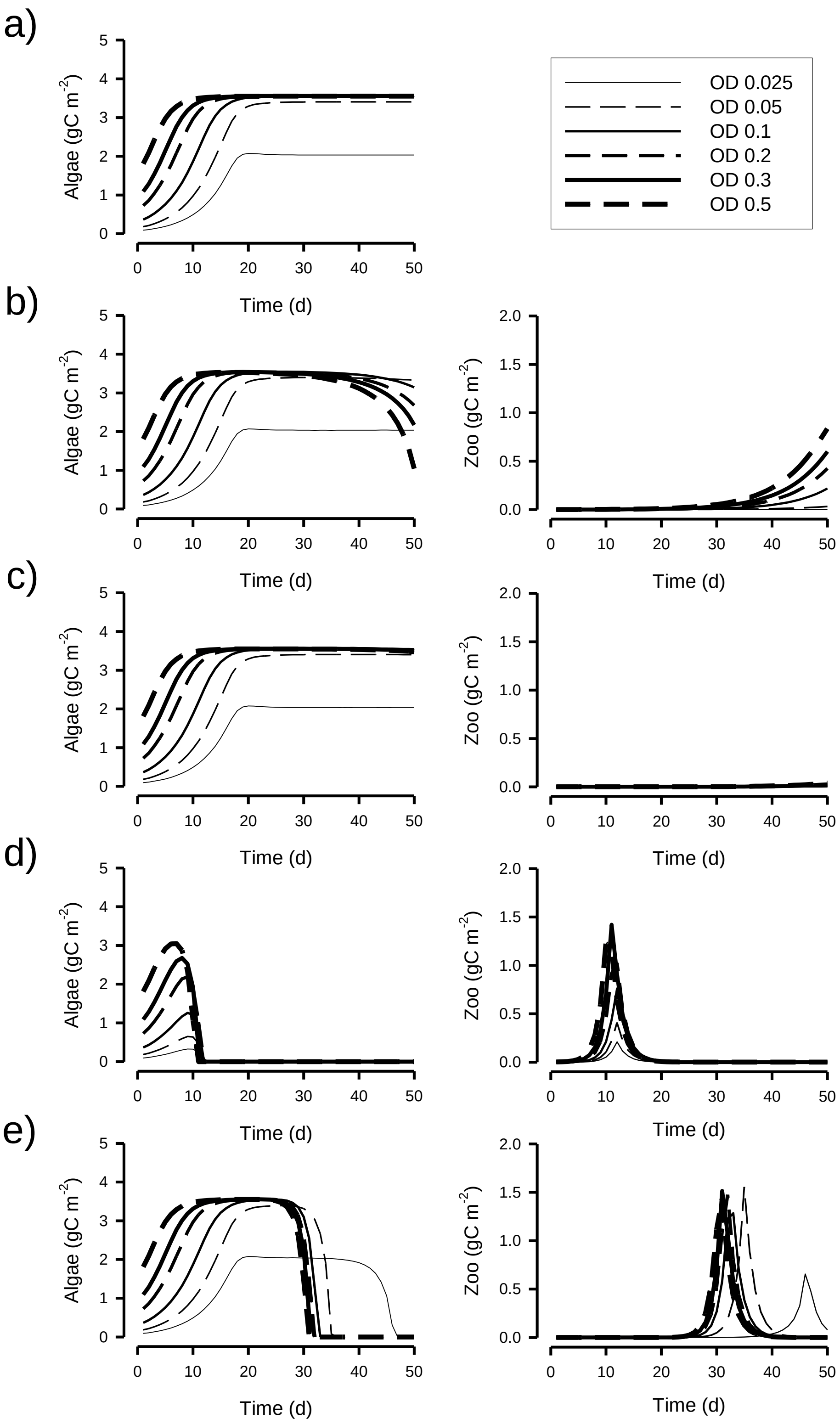

Fig. S3. Areal biomass, as Fig.S1, at 6 different operation depths (OD; 0.025 – 0.5 m), with a supply nutrient mole ratio N:P of 16, but a dilution rate of 0.5 d^{-1}. a) No contamination. b) Contamination at 0d. c) Contamination at 20d. d) Contamination at 0d with fast growing zooplankton. e) Contamination at 20d with fast growing zooplankton.

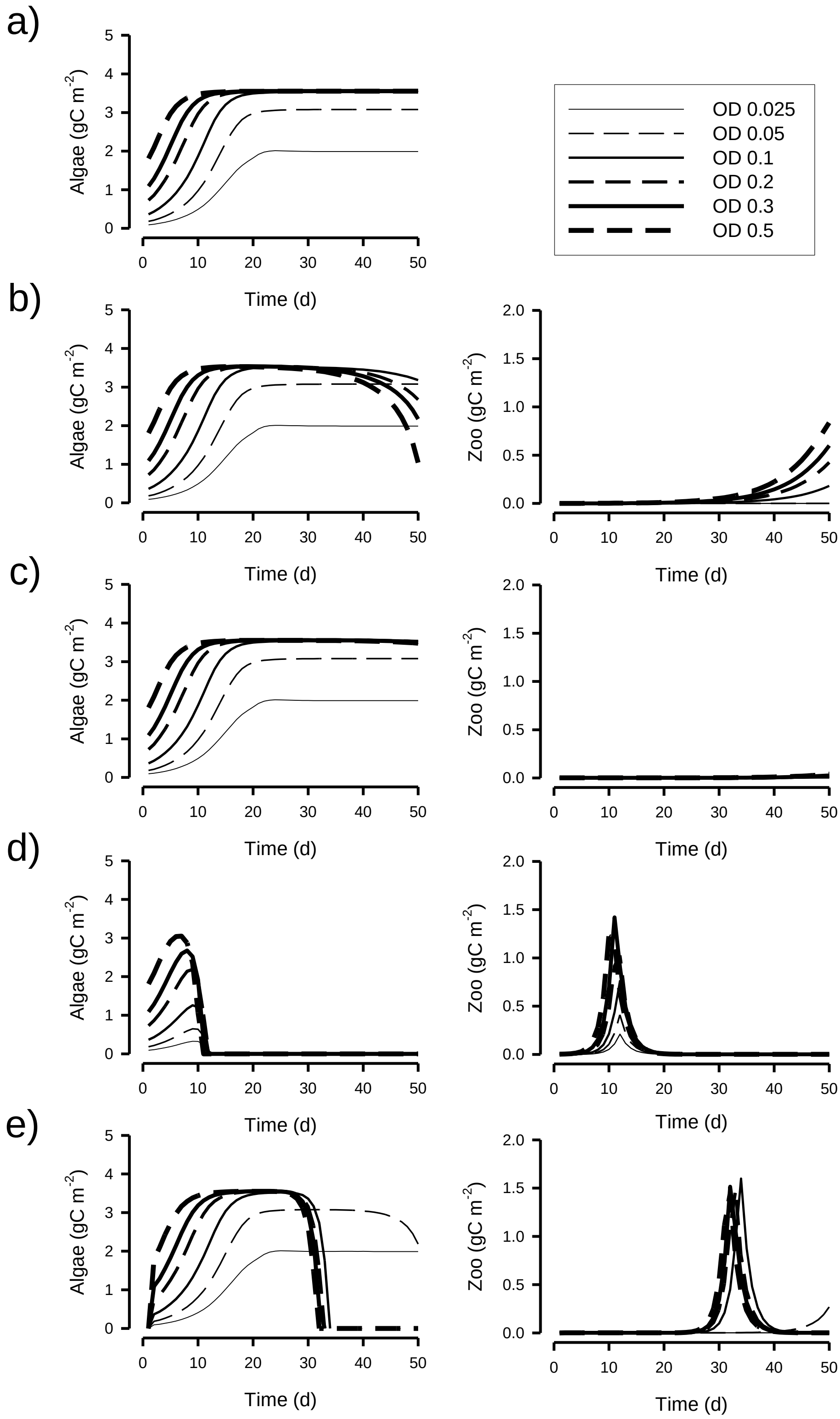

Fig. S4. Areal biomass, as Fig.S1, at 6 different operation depths (OD; 0.025 – 0.5 m), but with a supply nutrient mole ratio N:P of 32, and a dilution rate of 0.5 d^{-1}. a) No contamination. b) Contamination at 0d. c) Contamination at 20d. d) Contamination at 0d with fast growing zooplankton. e) Contamination at 20d with fast growing zooplankton.

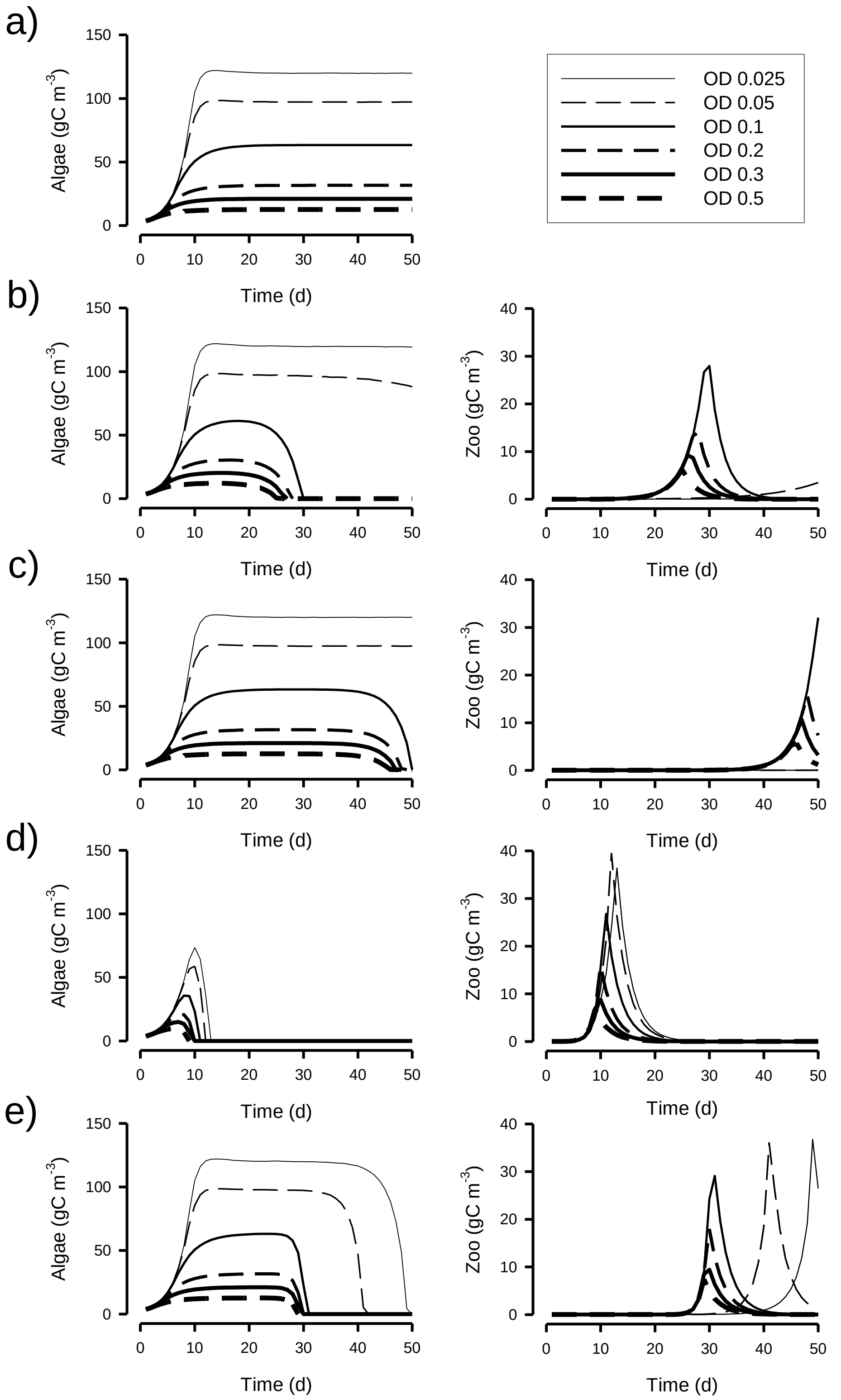

Fig. S5. Volumetric biomass of algae and a zooplankton contaminant grown at 6 different operational depths (OD; 0.025 – 0.5 m), with the supply nutrient mole ratio N:P at 16 and a dilution rate of $0.3d^{-1}$. Cf. Fig. 2 for biomass. a) No contamination. b) Contamination at 0d. c) Contamination at 20d. d) Contamination at 0d with fast growing zooplankton. e) Contamination at 20d with fast growing zooplankton.

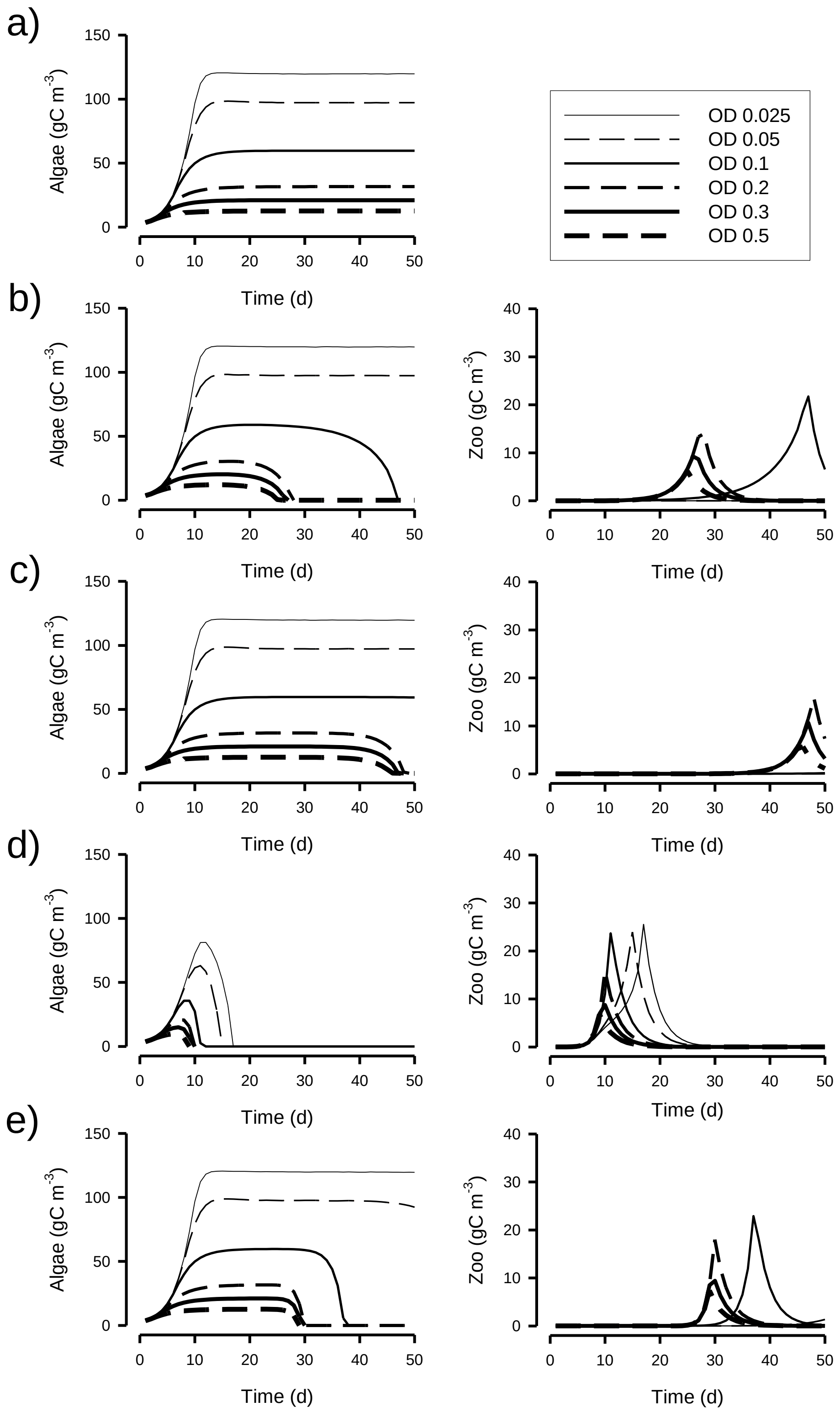

Fig. S6. Volumetric biomass, as Fig.S5 at 6 different operation depths (OD; 0.025 – 0.5 m), with a dilution rate of 0.3 d^{-1}, but with a supply nutrient mole ratio N:P of 32. Cf. Fig. 3 for biomass. a) No contamination. b) Contamination at 0d. c) Contamination at 20d. d) Contamination at 0d with fast growing zooplankton. e) Contamination at 20d with fast growing zooplankton.

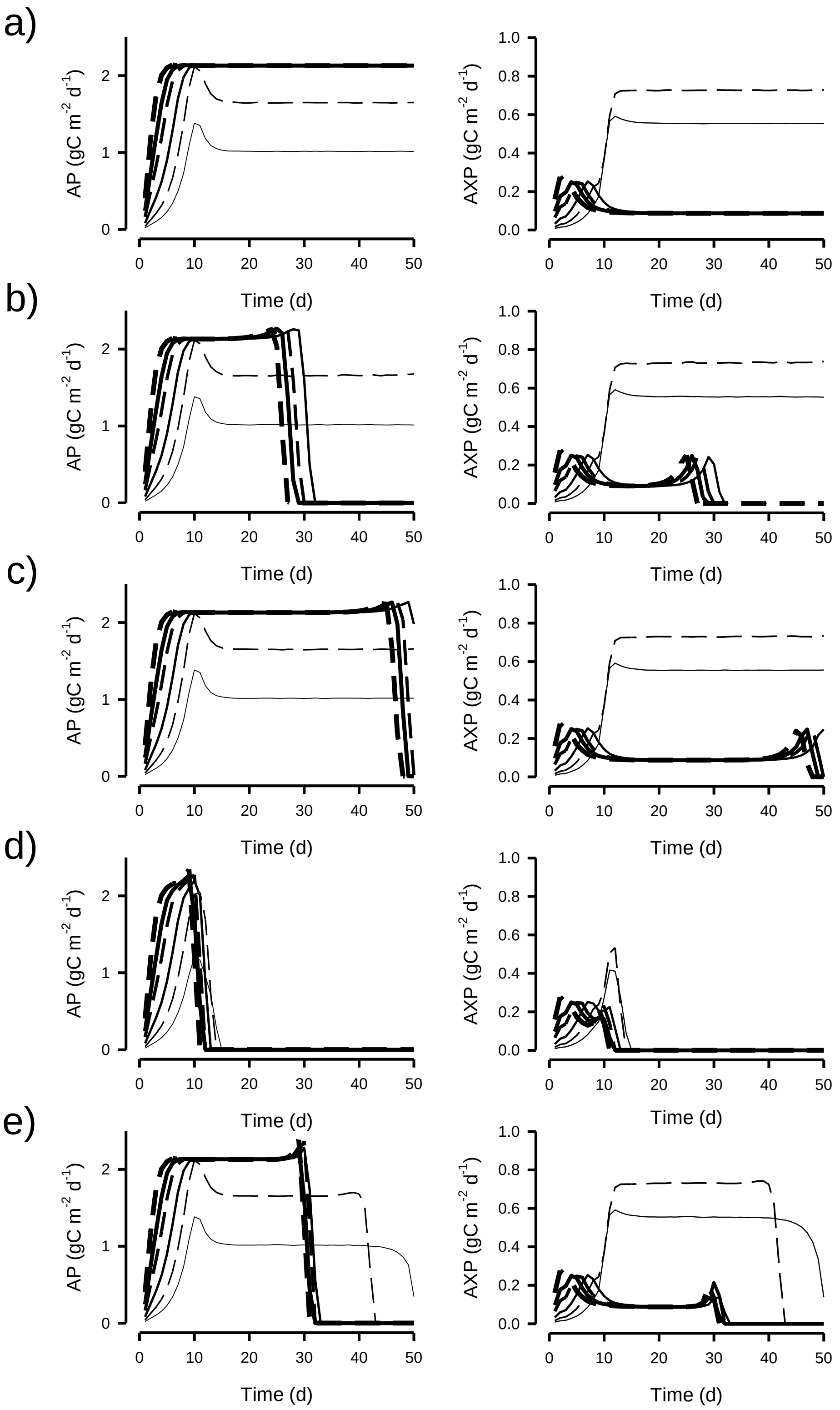

Fig. S7. Areal production of biomass (AP) and biofuels (AXP) at 6 different operational depths (OD; 0.025 – 0.5 m; see line style definitions in Fig. S6), with the supply nutrient mole ratio N:P at 16 and a dilution rate of 0.3d^{-1}. Cf. Fig. 2 for biomass. a) No contamination. b) Contamination at 0d. c) Contamination at 20d. d) Contamination at 0d with fast growing zooplankton. e) Contamination at 20d with fast growing zooplankton.

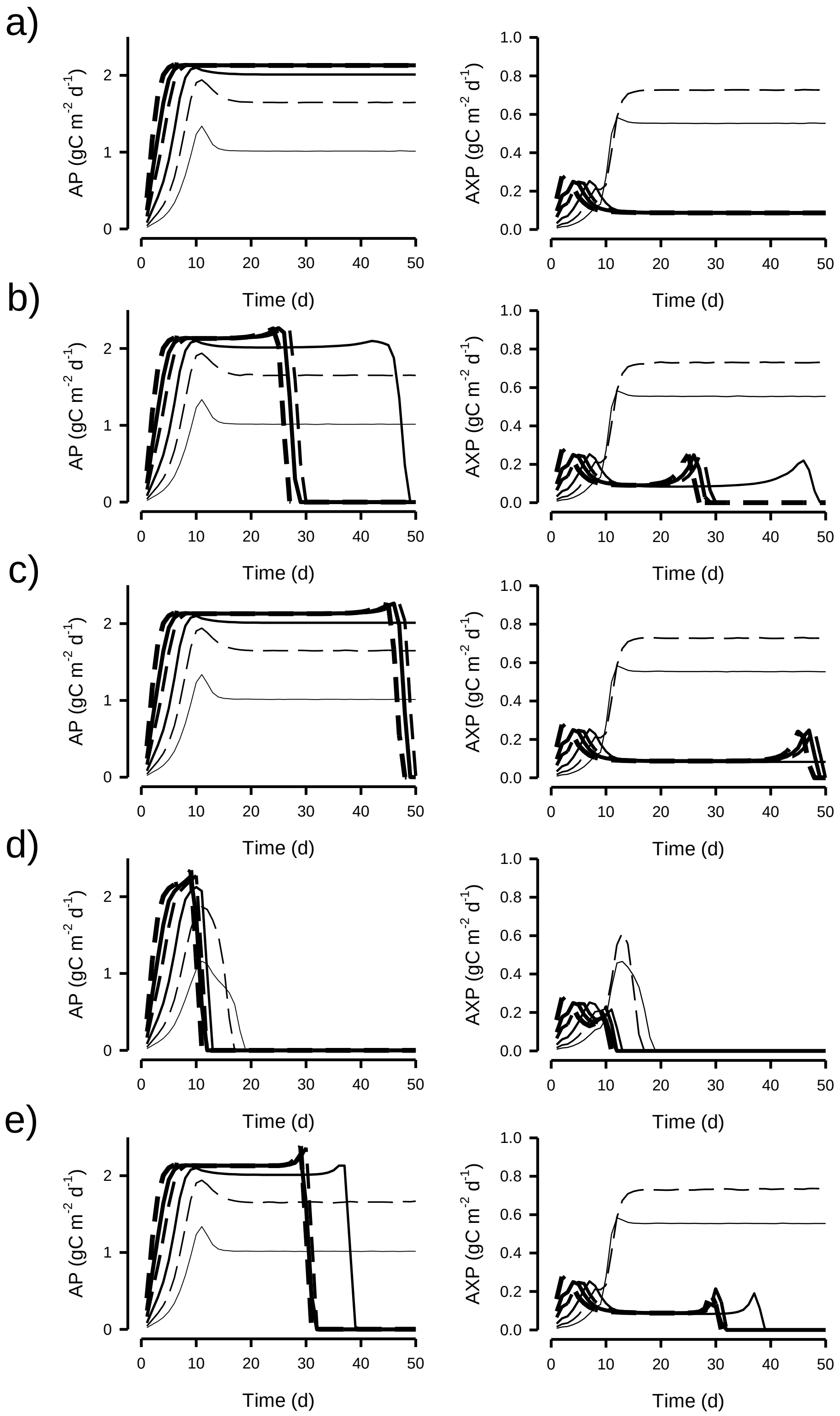

Fig. S8. Areal production of biomass (AP) and biofuels (AXP), as Fig. S7, with a dilution rate of 0.3 d^{-1}, but with a supply nutrient mole N:P of 32. Cf. Fig. 3 for biomass; see line style definitions in Fig. S6. a) No contamination. b) Contamination at 0d. c) Contamination at 20d. d) Contamination at 0d with fast growing zooplankton. e) Contamination at 20d with fast growing zooplankton.

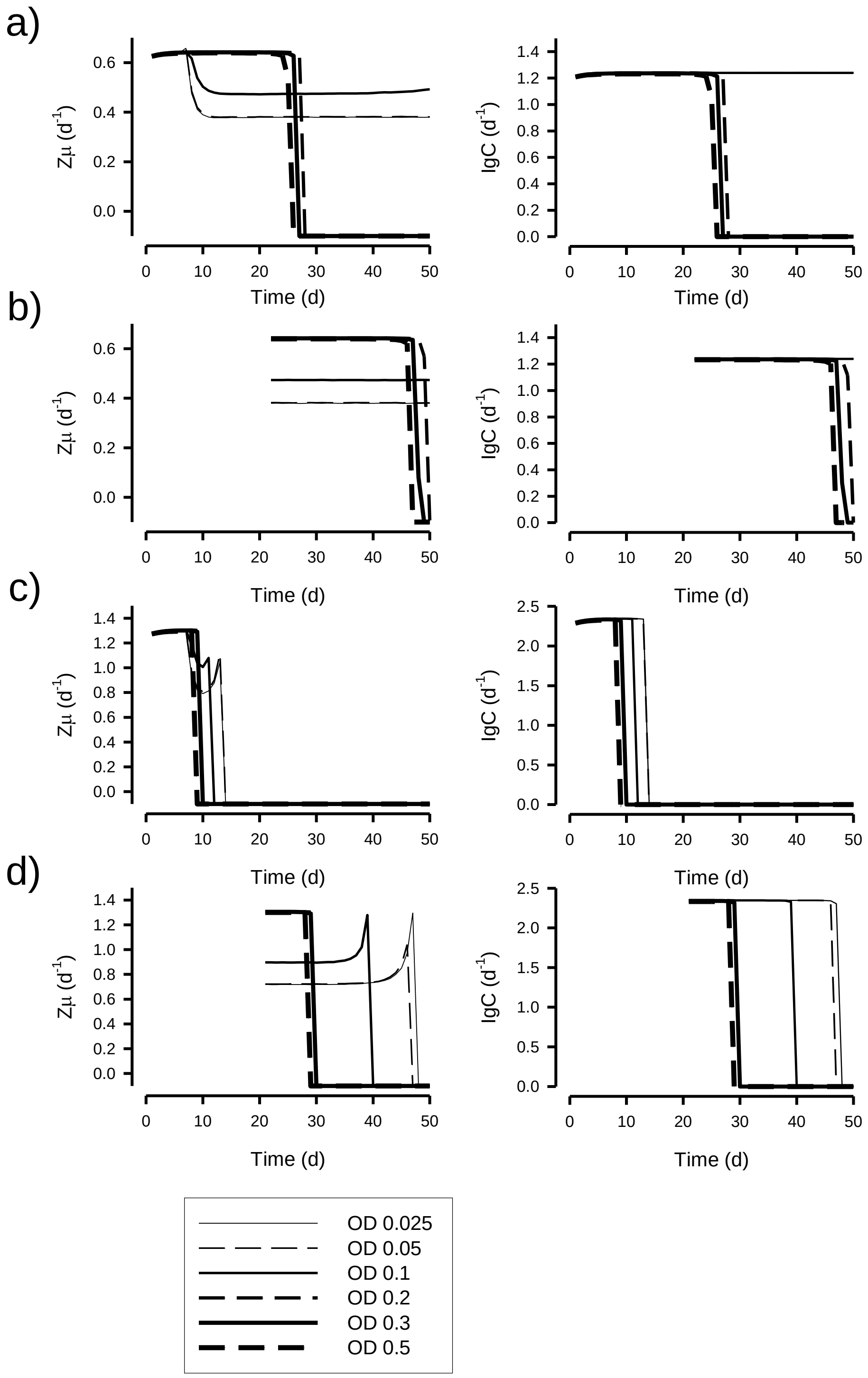

Fig. S9. Zooplankton growth rate (Zμ) and ingestion rate (IgC) at 6 different operational depths (OD; 0.025 – 0.5 m), with the supply nutrient mole ratio N:P at 16 and a dilution rate of $0.3d^{-1}$. Cf. Fig. 2 for biomass. a) Contamination at 0d. b) Contamination at 20d. c) Contamination at 0d with fast growing zooplankton. d) Contamination at 20d with fast growing zooplankton.

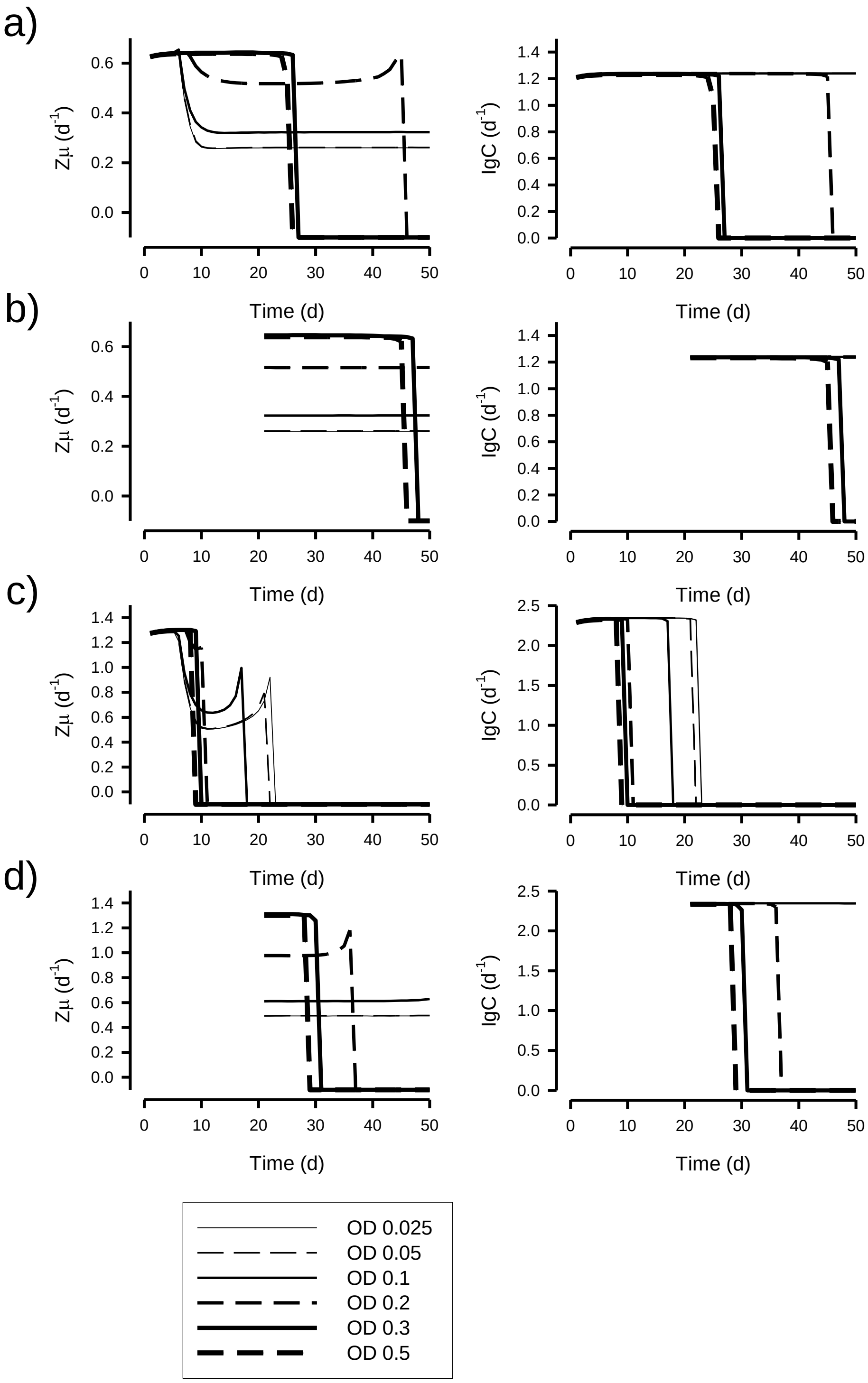

Fig. S10. Zooplankton growth rate (Zμ) and ingestion rate (IgC), as Fig. S9 at 6 different operation depths (OD; 0.025 – 0.5 m), with a dilution rate of 0.3 d^{-1}, but with a supply nutrient mole N:P of 32. Cf. Fig. 3 for biomass. a) Contamination at 0d. b) Contamination at 20d. c) Contamination at 0d with fast growing zooplankton. d) Contamination at 20d with fast growing zooplankton.

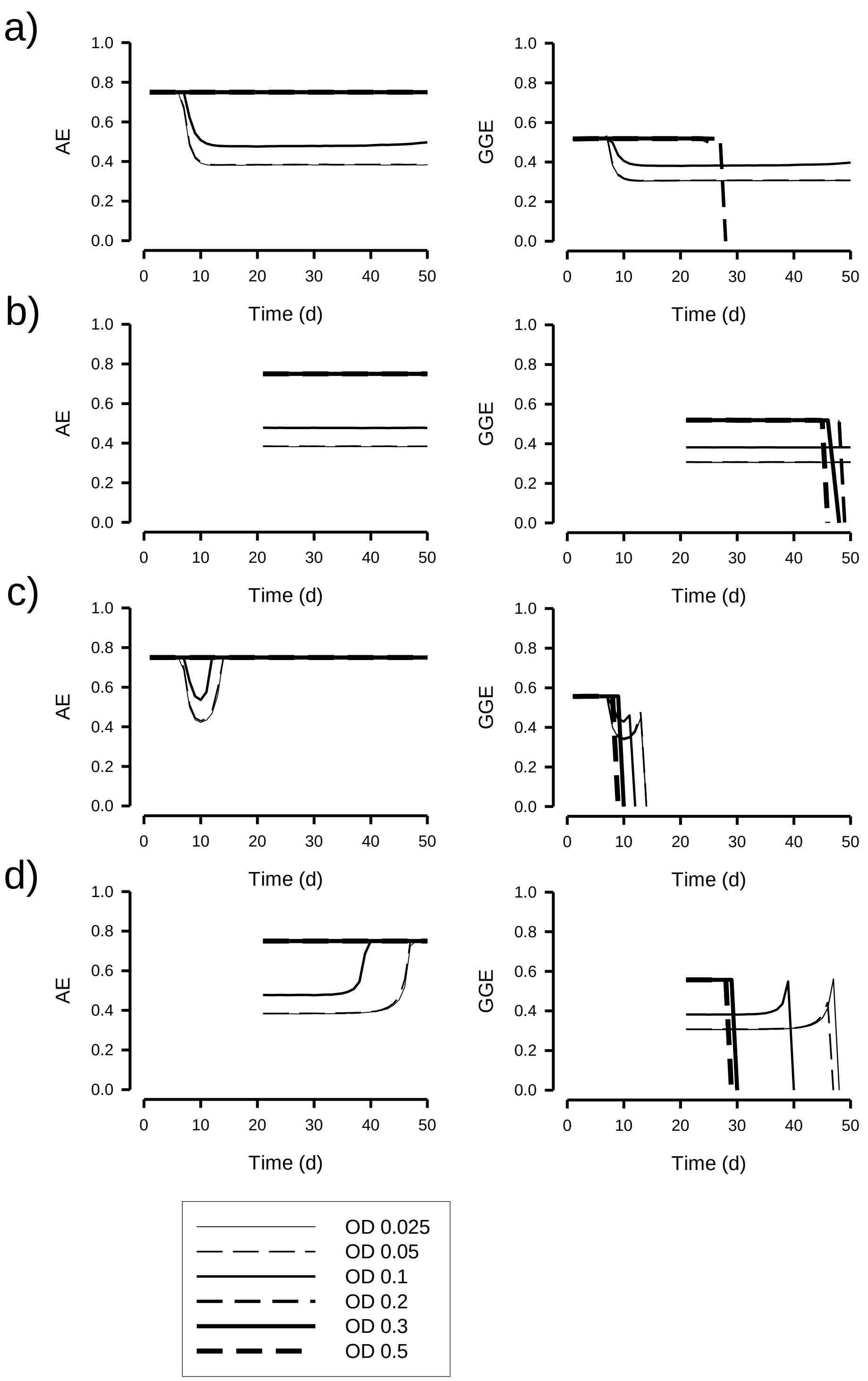

Fig. S11. Zooplankton assimilation efficiency (AE) and gross growth efficiency (GGE) at 6 different operational depths (OD; 0.025 – 0.5 m), with the supply nutrient mole ratio N:P at 16 and a dilution rate of $0.3d^{-1}$. Cf. Fig. 2 for biomass. a) Contamination at 0d. b) Contamination at 20d. c) Contamination at 0d with fast growing zooplankton. d) Contamination at 20d with fast growing zooplankton.

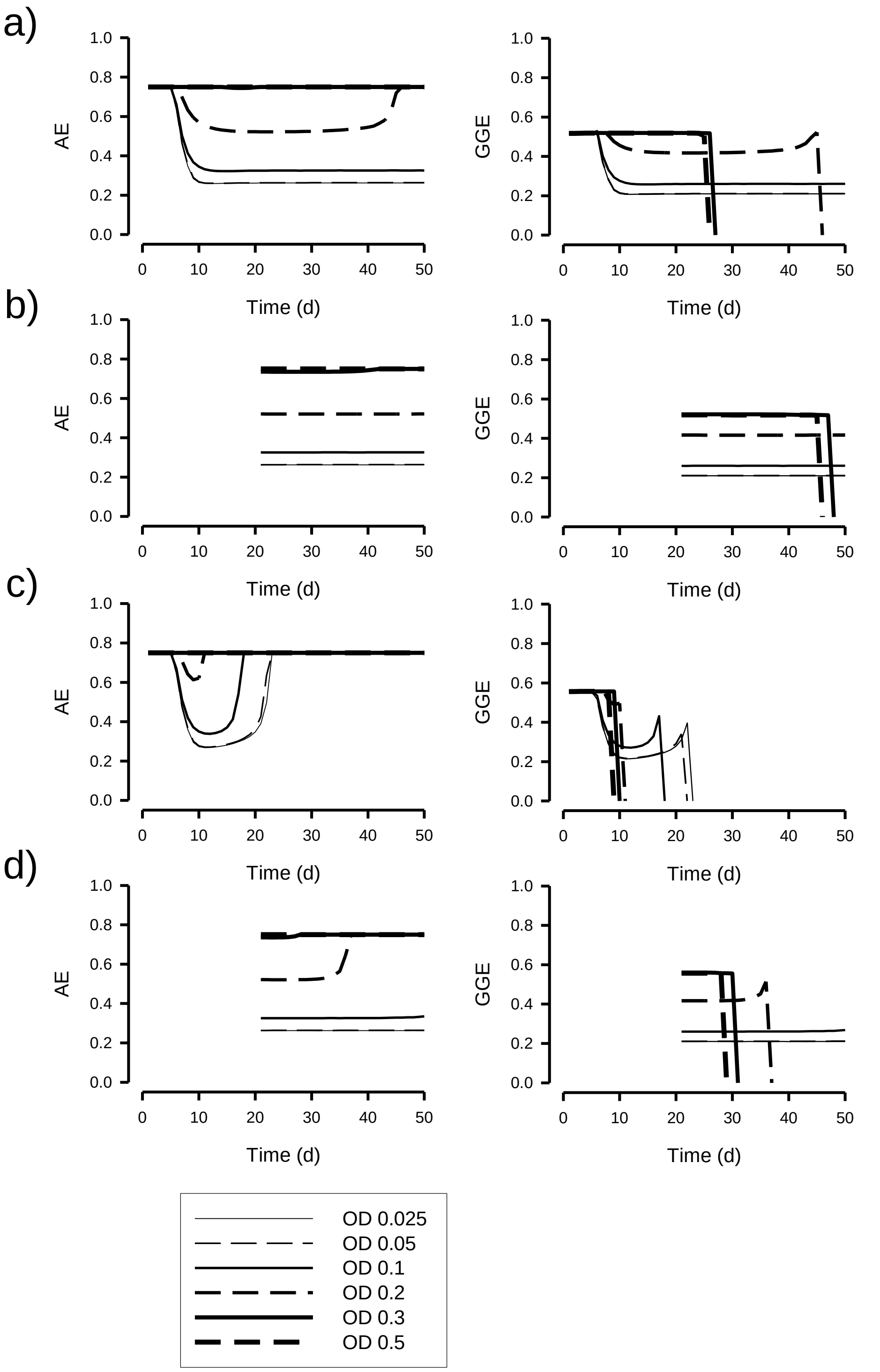

Fig. S12. Zooplankton assimilation efficiency (AE) and gross growth efficiency (GGE), as Fig. S11 at 6 different operation depths (OD; 0.025 – 0.5 m), with a dilution rate of 0.3 d^{-1}, but with a supply nutrient mole N:P of 32. Cf. Fig. 3 for biomass. a) Contamination at 0d. b) Contamination at 20d. c) Contamination at 0d with fast growing zooplankton. d) Contamination at 20d with fast growing zooplankton.
